# Advanced Technologies for Wastewater Treatment: Graphene-Based Catalysts

**DOI:** 10.3390/molecules30163405

**Published:** 2025-08-18

**Authors:** Justine Elgoyhen, Radmila Tomovska

**Affiliations:** 1 POLYMAT and Department of Applied Chemistry, Faculty of Chemistry, University of the Basque Country UPV/EHU, Avda Tolosa 72, 20018 Donostia-San Sebastian, Spain; justine.elgoyhen@ehu.eus; 2 IKERBASQUE, Basque Foundation for Science, Plaza Euskadi 5, 48009 Bilbao, Spain

**Keywords:** reduced graphene oxide, aerogel, xerogel, hydrogel, water remediation, organic dyes

## Abstract

This short review provides a focused overview of recent advances in catalytic systems for water purification, with particular attention to photocatalysis, Fenton-like processes, and biocatalysis. While not intended as a comprehensive survey, this review is grounded primarily in recent work from our research group, supported by comparisons with relevant studies from the broader literature. Emphasis is placed on the role of graphene-based materials, particularly aerogels, hydrogels, and xerogels, as functional platforms for catalytic nanoparticles inclusion and enzyme immobilization. This review aims to highlight key insights, practical limitations, and emerging strategies to improve catalyst reusability, stability, and scalability for real-world water treatment applications.

## 1. Introduction

Large-scale industrial activities, which continue to grow, pose increasing threats to both the environment and human health. Among these, the textile industry is particularly harmful to water quality due to the extensive use of various organic dyes. These dyes are used in large quantities to color textiles and are often discharged directly into rivers. This not only straightforwardly impacts aquatic flora and fauna but also indirectly degrades soil quality and contaminates agricultural products, leading to adverse effects on human health.

This situation has prompted numerous studies aimed at developing technologies that both prevent water pollution by organic dyes [1] and offer environmentally friendly, cost-effective methods to remove pollutants already present in water sources. Given the high levels of existing environmental contamination, a collaborative effort between industry and academia has emerged to explore effective strategies for wastewater treatment. As a result, a variety of physical, chemical, and biological approaches have been developed and, more recently, critically reviewed [2].

These methods should ideally be implemented before the release of polluted and highly concentrated wastewater into the environment, in order to protect both ecosystems and human health. Although physical methods—such as adsorption, extraction, precipitation, and filtration—can be highly effective in removing organic dyes [3], their primary drawback is that they merely transfer the pollutants rather than eliminate them, necessitating further post-treatment to achieve complete purification. In contrast, various chemical methods allow for the direct removal of organic dyes from water, breaking them down into less toxic intermediates or fully mineralizing them into carbon dioxide and water [4]. The development of nanomaterials has been a breakthrough in water purification technologies through sophisticated catalyst systems that provide a base for advanced processes of the capture of organic dyes from water and their degradation to complete mineralization. Numerous advanced oxidation processes have been developed based on the Fenton reaction [5,6], ozonation [7,8], or, more recently, sonolysis [9,10] or radiolysis [11,12]. However, the lack of efficiency in such processes has resulted in their combination with heterogeneous catalysis-based processes. Such approaches stand out as cost-effective and energy-efficient solutions that are successful in removing a wide range of organic pollutants, including organic dyes.

Based on heterogeneous catalysis, different advanced oxidation technologies have been developed, in which radicals that induce the oxidative-degradation processes of water pollutants may be created thermally, chemically, or by UV/Vis light [13].

Recently, numerous review works have been published, summarizing the advances in the field of catalytic degradation of organic dyes from wastewater. Nevertheless, most of these works summarize advanced oxidation processes based on heterogeneous photocatalysis [13,14,15].

The primary objective of this work is to provide a comprehensive overview of the research conducted by our group over the past fifteen years, centered on the development of three-dimensional (3D) hydrogel, xerogel, and aerogel architectures composed of reduced graphene oxide (rGO) platelets. These graphene-based porous frameworks have been investigated as versatile and efficient substrates for the immobilization of a wide range of catalysts, including photocatalysts, bio-enzymatic catalysts, and Fenton-type catalysts, with a particular emphasis on their application in the degradation of organic dyes and water purification.

This study not only consolidates our group’s contributions in the field but also places them in context by comparing our findings with similar studies reported in the literature. Through this comparative analysis, we highlight the unique advancements, innovations, and performance improvements achieved within our research program.

Importantly, to the best of the authors’ knowledge, this is the first comprehensive study to jointly examine and summarize the performance of different types of heterogeneous advanced oxidation processes (AOPs)—including photocatalysis, enzymatic oxidation, and Fenton-like reactions—all based on graphene-derived nanoporous 3D structures. By integrating these approaches into a unified framework, this work aims at providing a holistic perspective on the potential of graphene-based 3D materials as multifunctional platforms for water purification and the degradation of persistent organic pollutants.

## 2. Graphene-Structured Hydro-, Xero-, and Aerogels

Three-dimensional graphene nanostructures are usually synthesized by a self-assembly process induced by the chemical reaction of colloidal aqueous graphene oxide (GO) reduced using reducing agents [16]. During the reduction process, oxygen-containing functional groups (epoxy, hydroxyl, and carboxyl) on the GO platelets are removed, increasing the hydrophobicity of the sheets. This change in surface chemistry raises the interfacial tension between the rGO sheets and the surrounding aqueous phase, resulting in rGO sheets spontaneously self-assembling to minimize interfacial contact, leading to the formation of monolithic 3D structures with hierarchical porous morphology. In this process, a type of graphene hydrogel is formed, characterized by a three-dimensional network of reduced graphene oxide (rGO) sheets swollen with water. The underlying mechanism responsible for this nanostructuring, including the generation of pores of various sizes, has been previously elucidated [16].

Once the hydrogel is formed, it can be converted into a solid nanostructured material through different drying techniques. The choice of drying method significantly influences the final textural, structural, and mechanical properties of the material. Drying at elevated temperatures typically results in a xerogel, which is a denser, less porous form [17]. In contrast, applying freeze-drying or supercritical drying preserves the delicate porous network, yielding an aerogel with ultralow density and high surface area [18].

Although this chemical reduction self-assembly route for producing 3D nanostructured graphene is among the most straightforward, energy-efficient, and time-saving strategies available, numerous alternative methodologies have been reported in the literature. Among the various approaches, the most commonly employed methods for producing three-dimensional graphene-based structures include the following:

(i) Hydrothermal reduction, which typically involves subjecting a graphene oxide (GO) dispersion to elevated temperatures—significantly higher than those used in conventional chemical reduction methods [19,20]. Under these conditions, GO undergoes thermal reduction and self-assembly simultaneously, resulting in the formation of a hydrogel with an interconnected porous network. This method is valued for its simplicity and scalability, although it generally requires longer reaction times and high-pressure vessels (autoclaves). In general, the produced structures are less mechanically stable than these produced by chemical reduction methods.
(ii) Chemical vapor deposition (CVD), a more sophisticated technique that requires specialized equipment capable of generating and controlling reactive gas-phase precursors [10,21]. In this process, graphene layers are deposited onto sacrificial templates, often metal foams or porous structures, that serve as scaffolds for the growth of the 3D architecture. After deposition, the template is typically removed via chemical etching, leaving behind a freestanding, continuous graphene framework. While CVD offers excellent control over structural uniformity and crystallinity, it is relatively complex, costly, and less energy-efficient compared to solution-based methods.

## 3. Graphene-Structured Biocatalysts

Biocatalysts are made of enzymes or proteins capable of catalyzing specific reactions. They have attracted considerable attention as sustainable and highly efficient technologies for water treatment. Unlike conventional chemical catalysts, biocatalysts can operate under mild conditions across a wide pH range. They exhibit high substrate affinity, enabling the degradation of pollutants or contaminants even at very low concentrations [22]. Water purification strategies can be finely tailored by selecting highly specific enzymes, such as perchlorate reductase [22,23], or more broadly acting enzymes like laccase, which can degrade a wide spectrum of contaminants [22,24,25].

Biocatalysts for water treatment can be broadly categorized into isolated enzymes and whole-cell systems or microorganisms, both of which can be applied in free or immobilized forms [22,25,26]. When immobilized on suitable support materials, enzymes and microorganisms typically demonstrate improved stability, resistance to environmental stress, and enhanced reusability compared to their free counterparts [26,27]. As a result, the integration of biocatalysts into engineered materials and supports has opened new opportunities for their application in water purification technologies.

Common immobilization supports include natural materials such as cellulose [28,29] and chitosan [29], synthetic polymers like polyurethane [30], inorganic matrices such as silica [31] and activated carbon [32], and nanostructured materials like graphene oxide (GO) [33,34], carbon nanotubes (CNTs) [35], and metal–organic frameworks (MOFs) [36]. Among these, GO has emerged as a particularly promising support material for biocatalysts due to its high density of functional groups, which offer highly tunable surface chemistry. GO can be readily modified and functionalized to form complex structures and enable efficient enzyme immobilization. In addition to its chemical versatility, GO’s platelet-like morphology provides a large surface area, flexibility, and excellent mechanical properties [17,27].

The use of GO nanosheets as immobilization supports for biocatalysts has been widely reported [33,34,37]. However, it is important to note that working with two-dimensional (2D) nanostructures at the nanoscale can pose challenges due to their tendency to aggregate in aqueous systems [34], which limits their scalability. Consequently, graphene-based hydrogels, xerogels, and aerogels have garnered increasing attention as immobilization platforms, offering high porosity and enzyme-loading capacity [17,18,38].

In our research (Rodriguez-Couto et al. [17]), reduced graphene oxide (rGO) hydrogels and xerogels were investigated as supports for the immobilization of *Trametes pubescens* (*T. pubescens*) and laccase production under semi-solid-state fermentation (SSSF) conditions. The rGO hydrogels were synthesized via the ascorbic acid (AsA)-mediated reduction of graphene oxide (GO), resulting in rGO platelets that are predominantly hydrophobic but retain some oxygen-containing groups, imparting amphiphilic properties. This amphiphilicity facilitates the self-assembly of rGO sheets into 3D porous nanostructures capable of swelling in water. By tuning synthesis parameters such as temperature and GO/AsA ratio, a variety of materials with different chemical compositions and physical properties were obtained. FTIR spectroscopy revealed that increasing either the temperature or AsA concentration led to greater incorporation of AsA into the final 3D structures, likely through hydrogen bonding between carboxylic groups on both AsA and rGO, forming supramolecular rGO/AsA complexes. These complexes contributed to improved structural integrity and physical characteristics of the hydrogels.

**Figure 1:**
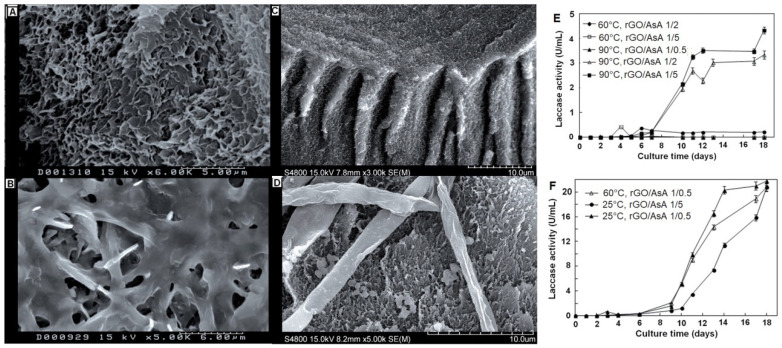
(**A**). rGO hydrogel; (**B**). rGO hydrogel with incorporated fungus; (**C**). rGO xerogel; (**D**) rGO xerogel with incorporated fungus; (**E**). laccase enzyme activity in hydrogel; (**F**). laccase enzyme activity in xerogels. Adapted with permission from Ref. [17]. Copyright 2014 John Wiley and Sons.

Scanning electron microscopy (SEM) confirmed the formation of twisted fiber-like structures (~5 µm in diameter) with an average pore size of ~1 µm, attributed to the hydrogen-bonded rGO/AsA networks. Interestingly, the SEM images showed consistent morphology across samples despite variations in their physical properties (Figure 1A,B) [39]. Xerogels were produced by drying the hydrogels at 45 °C overnight. The twisted porous architecture was largely retained, though with reduced dimensions and pore sizes (~0.3 µm) due to shrinkage during drying. FTIR analysis indicated that xerogels lacked AsA, resulting in distinct chemical structures compared to hydrogels. Both rGO hydrogels and xerogels proved effective as inert supports for fungal colonization. The SEM images in Figure 1C,D show fungal hyphae well integrated into the porous matrix, with xerogels supporting visibly thicker and morphologically distinct hyphae, likely due to their smaller pore sizes, the absence of AsA, and higher hydrophobicity, conditions that enhanced fungal growth and enzyme production. Notably, the xerogel synthesized at 25 °C with a GO/AsA ratio of 1:0.5 achieved laccase activity of 20 ± 1 U/mL (Figure 1F), significantly surpassing that of hydrogels (~4.3 U/mL; Figure 1E) and traditional inert supports like polyurethane foam or stainless-steel sponge (~2–3 U/mL). The superior performance was attributed to the xerogel’s stable, hydrophobic 3D architecture, which promoted favorable fungal–material interactions and enzyme secretion. Graphene-based hydrogels and xerogels are effective as supports for biocatalytic systems such as *T. pubescens*. Their findings highlight the synergistic role of material morphology and chemical composition in enhancing fungal growth and laccase activity. These conclusions align with recent findings by Wu et al., who reported improved laccase immobilization and stability using highly porous bacterial cellulose aerogels [28].

Despite the excellent performance achieved, we noted practical challenges in handling and processing 3D graphene-based materials, highlighting a key limitation. To address issues of mechanical robustness, scalability, and cost-effectiveness, the integration of polymer chemistry to form composite 3D matrices has been proposed as a promising strategy. In our related study, Ormategui et al. [18] developed a simple, eco-friendly method for fabricating nanobiocatalysts by immobilizing laccase onto self-assembled 3D composite hydrogels composed of reduced graphene oxide (rGO) reinforced with polymer nanoparticles. These hydrogels were synthesized by reducing GO with ascorbic acid (AsA) in the presence of poly(methyl methacrylate-co-butyl acrylate) (poly(MMA/BA)) polymer colloidal particles. Initially, the polymer nanoparticles adsorbed onto GO sheets via hydrogen bonding to form hybrid GO nanoplatelets, which were subsequently reduced with AsA at 90 °C. The resulting rGO–polymer hybrid nanoplatelets self-assembled into porous, water-swollen 3D networks, and the incorporation of polymer into the hydrogel was confirmed by FTIR spectroscopy.

Consistent with earlier findings reported by Rodriguez-Couto et al. [17], mechanical performance improved significantly with higher AsA concentrations at a constant polymer content. Notably, the hydrogel synthesized with an AsA/GO ratio of 2 exhibited a storage modulus of 513 kPa, surpassing conventional self-assembled polymeric [35,36] and graphene-based [18,37] hydrogels by 1–3 orders of magnitude. This enhancement is attributed both to increased AsA integration within the supramolecular structure and to pH-dependent polymer incorporation. At higher AsA concentrations (i.e., lower pH), the protonation of GO functional groups promotes hydrogen bonding with the polymer, leading to greater polymer integration and improved stiffness.

Variations in polymer particles loading within rGO structures (rGO/polymer weight ratios of 1/0.8 and 1/2.4), monomer composition (MMA/BA ratios of 50/50 and 70/30), and polymer particle surface functionalization (hydroxyl or bromo groups) enabled the fine-tuning of the hydrogel’s mechanical properties and morphology. Both rGO/polymer ratios led to significant increases in the storage modulus compared to neat rGO hydrogels, with 1/0.8 identified as the optimal ratio. Excess polymer led to reduced mechanical strength, likely due to the formation of larger hybrid platelets and increased pore size, as confirmed by SEM. Hydrogels incorporating Br- and OH-functionalized copolymers were also compared. The Br-functionalized copolymer (70/30 MMA/BA) exhibited superior stiffness, while the OH-functionalized variant (50/50 MMA/BA) showed a lower mechanical performance, attributed to the higher content of soft BA monomer (Tg = −54 °C). These results underscore the strong influence of polymer composition on hydrogel mechanics and the versatility of the synthetic approach for tailoring material properties.

The laccase enzyme was covalently immobilized onto the hydrogels via simple contact at room temperature, achieving immobilization yields between 58.5% and 75.9%. The resulting biocatalysts efficiently degraded Remazol Brilliant Blue R (RBBR), with the complete decolorization of concentrated dye solutions maintained over four consecutive treatment cycles. These results demonstrate the potential of reinforced graphene-based hydrogels as robust, reusable supports for biocatalysis. Moreover, according to Zucconi’s test, the obtained values for GI of 100% demonstrated that the composite material presents no phytotoxicity, and the same is true of the RBBR solution after Lacasse treatment.

A similar strategy was recently reported by Zhang et al. [38], who synthesized lignin-reinforced graphene aerogels via a one-step hydrothermal process for lipase immobilization. The inclusion of lignin significantly enhanced both mechanical strength and enzyme immobilization efficiency. The immobilized lipase maintained over 94% of its initial activity after four catalytic cycles, indicating excellent stability and reusability.

It is worth highlighting that our strategy for synthesizing composite polymer/rGO hydrogels is distinct, as it performs the reduction of graphene oxide (GO) within aqueous colloidal dispersions of polymer nanoparticles, which serve as a matrix for the self-assembly process. This approach enables direct contact between the polymer nanoparticles and GO sheets prior to reduction, facilitating supramolecular interactions and the formation of hybrid nanoplatelets—graphene sheets decorated with polymer particles. During the ascorbic acid-mediated reduction, these hybrid nanoplatelets spontaneously organize into composite 3D porous networks, highly swollen with water—namely, graphene/polymer hydrogels. Depending on the drying conditions applied, these hydrogels can be further converted into xerogels or aerogels.

Together, these findings illustrate that advanced graphene-based composites offer a powerful platform for high-performance, recyclable biocatalyst systems, suitable not only for water purification but also for broader applications, including photoactive biomaterials [17].

## 4. Graphene Aerogel-Structured Photocatalysts

Graphene platelets, characterized by their exceptionally high specific surface area and outstanding physicochemical properties, have emerged as promising substrates for the immobilization of photocatalytic nanoparticles. Their large surface area facilitates a high loading capacity for nanoparticles, ensuring uniform dispersion and strong interfacial contact, which are critical for enhancing photocatalytic activity. Furthermore, graphene’s excellent electrical conductivity enables efficient charge transfer, contributing to a reduction in electron–hole recombination rates, a key limitation in many photocatalytic systems. The integration of nanoparticles with graphene not only improves photocatalytic efficiency by narrowing the band gap and increasing light absorption but also offers practical benefits such as improved structural stability, reusability, and ease of separation from treated media.

Despite these advantages, the use of powdered graphene-based materials in heterogeneous photocatalysis has encountered several challenges that limit their practical implementation. Principal among these are difficulties in the efficient recovery and reuse of photocatalyst nanoparticles, significant material losses during recycling processes, and concerns regarding the environmental and biological safety of residual nanoparticles, which may pose cytotoxic risks to human or animal health. To overcome these limitations, researchers have shifted focus toward the development of macroscopic, three-dimensional (3D) graphene-based architectures, particularly graphene aerogels with hierarchical nanoporous structures. These aerogels combine the intrinsic benefits of graphene with improved mechanical integrity, high porosity, and large accessible surface areas. Such features allow for better diffusion of reactants, enhanced light harvesting, and more efficient separation and recyclability of the photocatalyst. As a result, 3D graphene aerogels have demonstrated superior performance in the photodegradation of organic pollutants, including synthetic dyes and pharmaceuticals, under various irradiation conditions.

The promise shown by these advanced materials has led to a surge in research activity, with a rapidly growing number of publications exploring their synthesis, functionalization, and application in environmental remediation. This growing body of literature has been extensively reviewed in several recent comprehensive articles, such as the work by Long et al. [14], Lu et al. [15], and Guo et al. [6] which systematically evaluates the progress, challenges, and future prospects of 3D graphene-based photocatalytic systems.

Semiconductor nanoparticles with photocatalytic properties are typically integrated into graphene aerogel nanostructures spontaneously during the chemical reduction process used to synthesize the aerogels. In this in situ approach, nanoparticles are formed directly from appropriate precursors within the evolving aerogel matrix [14]. In most cases, various metal oxide nanostructures have been employed for this purpose. Studies have shown that these composite heterojunction photocatalysts exhibit a more refined crystalline architecture with reduced defect density, which significantly enhances their photocatalytic performance under light irradiation. The intrinsically high surface area of graphene aerogels further contributes to this effect by enabling greater pollutant adsorption, facilitating more efficient mass transfer, extending light absorption capacity, and promoting effective charge separation.

Titanium dioxide (TiO_2_) nanoparticles represent one of the most extensively investigated photocatalysts, mainly in combination with graphene aerogels. In particular, the research group led by Nieu reported a significant reduction in the band gap of TiO_2_ from 3.2 eV to approximately 2.63 eV upon incorporation into the graphene aerogel matrix, resulting in the near-complete removal of Methylene Blue (MB) from aqueous solutions [40]. The remarkable photocatalytic efficiency of TiO_2_–graphene aerogel composites has been further demonstrated across a range of organic dyes, including Methylene Blue (MB), Rhodamine B (RhB), and Methyl Orange (MO) [30,31,32,33,34,35,36,37,38,39,40,41,42]. Despite the consistently high decolorization performance reported in these studies, the underlying photodegradation mechanisms and the extent of mineralization were not thoroughly investigated. Consequently, the identification and analysis of intermediate or final degradation products remain absent, limiting a comprehensive understanding of the photocatalytic process and its environmental implications.

**Figure 2:**
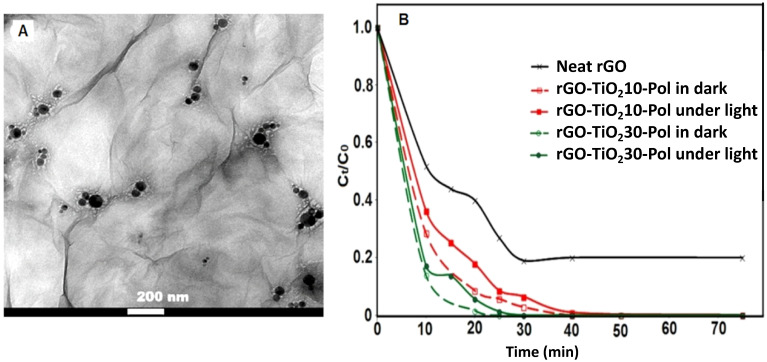
(**A**). TEM image of rGO hybrids with incorporated manganese-doped TiO_2_ nanoparticles. (**B**). Kinetics of MB degradation in aqueous solution by hybrids with different TiO_2_ amounts (10 or 30%) and in different conditions (in dark or under visible light) Adapted with permission from Ref. [43]. Copyright 2020 John Wiley and Sons.

In our recent study, manganese-doped TiO_2_ nanoparticles, exhibiting strong visible light absorption, were successfully integrated into a monolithic aerogel framework composed of reduced graphene oxide (rGO) [43]. The TEM image shown in Figure 2B demonstrates the inclusion of the nanoparticles onto the rGO sheets. The photocatalytic performance of these composite structures was evaluated for the degradation of MB in aqueous solution under visible light irradiation. To enhance the mechanical integrity of the monoliths, polyurethane/acrylic colloidal nanoparticles were incorporated into the rGO network during the chemical reduction process, which was initiated using ascorbic acid. This synthetic strategy yielded a mesoporous structure with a complex morphology and a specific surface area of about 138 m^2^/g. A notable red shift in the absorption maximum was observed, likely attributed to the strong interfacial interactions and bonding among rGO, the polymeric phase, and TiO_2_ nanoparticles. Furthermore, the optical band gap of the material was reduced to approximately 2.8 eV, enhancing visible-light-driven photocatalytic activity. As a result, the system exhibited outstanding performance, achieving both the complete adsorption and total degradation of MB in very short time, in less than 30 min (Figure 2B). Crucially, and in contrast to much of the current state of the art, our work extended beyond dye removal efficiency by investigating the degradation pathway using matrix-assisted laser desorption/ionization time-of-flight mass spectrometry (MALDI-TOF-MS). The analysis confirmed that MB degradation proceeded via the generation of hydroxyl (•OH) radicals. Detected degradation products included compounds containing one to three aromatic rings, alongside small, fully oxidized species such as H_2_O, CO_2_, NO_3_^−^, and SO_4_^2−^. The presence of these end-products is indicative of the near-complete mineralization of the dye, underscoring the efficiency and environmental relevance of the developed photocatalyst.

In the ongoing activities of enhancing photocatalytic efficiency, researchers have frequently explored the incorporation of TiO_2_ with other metal oxides or inorganic materials. For instance, prior to integration into graphene aerogels, TiO_2_ was combined with zinc oxide (ZnO), resulting in a marked improvement in photocatalytic performance compared to systems composed solely of TiO_2_ and graphene aerogels [44]. In this study, the degradation of MO and RhB in aqueous solution was investigated, and the photocatalytic efficiency was further validated through the identification of photodegradation products using liquid chromatography–mass spectrometry (LC-MS). Unlike the mechanism observed with TiO_2_ nanoparticles embedded in graphene aerogels, where hydroxyl radicals (•OH) predominantly drive the degradation, the use of TiO_2_/ZnO composite nanoparticles within the aerogel matrix revealed that superoxide radicals (•O_2_^−^) played the main role in the decomposition of the dye molecules.

Within our research group, particular attention has been directed toward tailoring the morphology of catalytic nanoparticles as a means to optimize the overall photocatalytic behavior of the resulting composite structures [45]. To this end, two-dimensional (2D) nanostructures of various metal oxides and inorganic semiconductors, including MoS_2_, WS_2_, GaN, CeO_2_, CdS, and ZnO, were synthesized through the sono-exfoliation of their respective bulk materials. These 2D materials were subsequently incorporated into reduced graphene oxide (rGO) aerogels to fabricate hybrid composite photocatalysts.

The central hypothesis guiding this work was that intimate 2D–2D interfacial contact between the exfoliated metal oxide nanosheets and rGO nanoplatelets would significantly enhance photocatalytic activity. This architecture creates an extensive interfacial area, which promotes stronger interphase interactions, more effective charge carrier separation, and a subsequent increase in photocatalytic performance. Similarly, as in previous works, in this case colloidal methacrylate particles were also added to these complex-morphology composite photocatalysts.

Indeed, the resulting composite catalysts exhibited a superior photocatalytic degradation of MB under UV light irradiation, outperforming both the pristine rGO aerogels and the individual 2D metal oxide nanostructures. Following the complete decolorization of MB, a MALDI-TOF-MS analysis of the treated aqueous solutions confirmed the occurrence of full mineralization. Particularly good performance was achieved by 2D WS_2_ nanoplatelets produced via the sono-exfoliation process. When embedded within the rGO aerogel framework, these WS_2_-based composites demonstrated the highest photocatalytic performance among the studied metal oxides. These advanced materials, featuring two-dimensional (2D) nanosheets of diverse photocatalysts immobilized within three-dimensional (3D) monolithic graphene aerogels, represent a highly versatile platform with broad application potential. Their unique architecture—combining high surface area, efficient light absorption, rapid charge separation, and robust structural integrity—positions them at the forefront of emerging technologies in environmental and energy-related fields.

The integration of 2D photocatalysts with graphene aerogels not only enhances photocatalytic efficiency through synergistic interfacial interactions but also opens pathways to designing lightweight, flexible, and scalable materials. This positions them as critical components in the development of sustainable, low-energy, and high-performance systems for addressing global challenges in clean energy, environmental remediation, and resource recovery.

Beyond their demonstrated effectiveness in water and air purification and CO_2_ photoreduction, these materials could play a pivotal role in hydrogen generation through photocatalytic water splitting, solar fuel production, and the degradation of persistent organic pollutants and pharmaceutical residues in wastewater. Additionally, their tailored optoelectronic properties make them suitable for use in sensors, photoelectrochemical cells, self-cleaning surfaces, and antimicrobial coatings.

## 5. Graphene Aerogel-Structured Fenton Catalysts

Advanced oxidation processes (AOPs) based on the Fenton reaction represent a pivotal strategy in environmental chemistry for the degradation of persistent pollutants, particularly organic dyes. These processes rely on the catalytic generation of highly reactive oxidative species, which facilitate the breakdown of contaminants. Among the various catalysts employed, magnetic nanoparticles (MNPs) are the most widely used due to their efficiency and ease of separation [46,47]. The underlying mechanism involves the redox cycling between Fe^3+^ and Fe^2+^ ions, which drives the decomposition of hydrogen peroxide (H_2_O_2_) into reactive hydroxyl radicals (•OH) [48].

However, traditional Fenton systems face significant limitations, such as the slow regeneration of Fe^2+^ and the formation of iron sludge, which hinders the overall efficiency and sustainability of the process. To address these challenges, magnetic nanoparticles are often immobilized on suitable supports. This immobilization serves two primary functions: it enhances the operational stability and recyclability of the catalyst, and it accelerates both the Fe^3+^/Fe^2+^ redox cycle and the generation of reactive radicals.

Graphene aerogels have emerged as highly effective support materials in heterogeneous Fenton systems due to their large surface area, excellent conductivity, and robust structural properties. Recent review articles have comprehensively summarized the advances in utilizing graphene aerogels as active supports, highlighting their critical role in improving the performance and applicability of Fenton-based AOPs [5].

To the best of the authors’ knowledge, only a single study has reported the application of a conventional (neat) Fenton reaction using a graphene-based catalyst for dye degradation. In that work, Su et al. employed α-FeOOH nanoparticles anchored onto a hybrid aerogel composed of graphene and carbon nanotubes (CNTs) to degrade the azo dye Orange II [49]. Although the system demonstrated high decolorization efficiency and the complete mineralization of degradation by-products, its practical applicability was limited by poor performance in repeated catalytic cycles, indicating suboptimal reusability.

To overcome the inherent limitations of the classical Fenton process, such as sluggish Fe^3+^ reduction and narrow pH range, light-assisted Fenton reactions, known as photo-Fenton processes, are commonly employed. These systems utilize UV or visible light to accelerate the Fe^3+^/Fe^2+^ redox cycling, thereby enhancing hydroxyl radical generation and overall reaction kinetics. Photo-Fenton processes have been widely applied for the oxidative degradation of various organic dyes, including RhB [50,51] and MO [52]. While these systems often achieve high degradation efficiency and improved catalyst stability over multiple cycles, several drawbacks remain. Reported issues include extended reaction times [51], low degradation rates [50], and the incorporation of potentially toxic or non-sustainable materials [52], which may raise environmental and scalability concerns.

**Figure 3:**
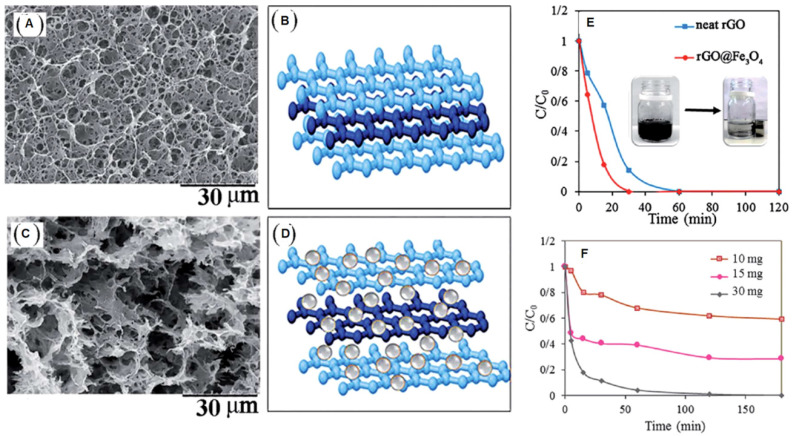
(**A**). SEM image of neat rGO aerogel structure. (**B**). Model of the stacked rGO sheets within the aerogel. (**C**). SEM image of the hybrid rGO/Fe_3_O_4_ aerogels. (**D**). Model of the hybrid rGO/Fe_3_O_4_ aerogels. (**E**). Kinetics of AR degradation in aqueous solution: comparison between neat rGO and hybrid aerogels. (**F**). Effect of the quantity of the hybrid aerogel on the AR degradation kinetics. Adapted with permission from Ref. [53]. Copyright 2020 Royal Society of Chemistry.

In our study [53], we conducted an in-situ synthesis of magnetic nanoparticles concurrently with the reduction-induced self-assembly of reduced graphene oxide (rGO) platelets, resulting in the formation of a three-dimensional (3D) monolithic aerogel structure with uniformly embedded magnetic nanoparticles (Figure 3). The incorporation of nanoparticles had a pronounced influence on the self-assembly process, significantly altering the textural characteristics of the aerogel—most notably by increasing the proportion of micropores. This effect can be attributed to the role of the nanoparticles as nanoscale spacers, which impeded the complete restacking of rGO sheets during the reduction process, thereby preserving porosity and enhancing surface area (as illustrated in Figure 3).

These nanostructured hybrid aerogels were employed as heterogeneous catalysts in Fenton reactions for the degradation of the organic azo dyes Acid Red (AR) [53] and Acid Green 25 (AG) [54] in aqueous media. In both cases, the complete degradation of the dyes was achieved, underscoring the exceptional catalytic performance of the materials. This high efficiency of AR degradation by Fenton reaction (Figure 3E) is ascribed to the formation of charge-transfer complexes between the magnetic nanoparticles and the rGO framework [53]. Such intimate interfacial bonding, achieved through the simultaneous reduction of graphene oxide and FeSO_4_·7H_2_O, likely facilitates more effective electron transfer processes that are critical for radical generation in the Fenton reaction [53]. Figure 3F shows the effect of the hybrid catalysts rGO/Fe_3_O_4_ loading on the decolorization process. Increasing the catalyst amount raises the process rate and makes possible the complete decolorization of the aqueous solution.

**Figure 4:**
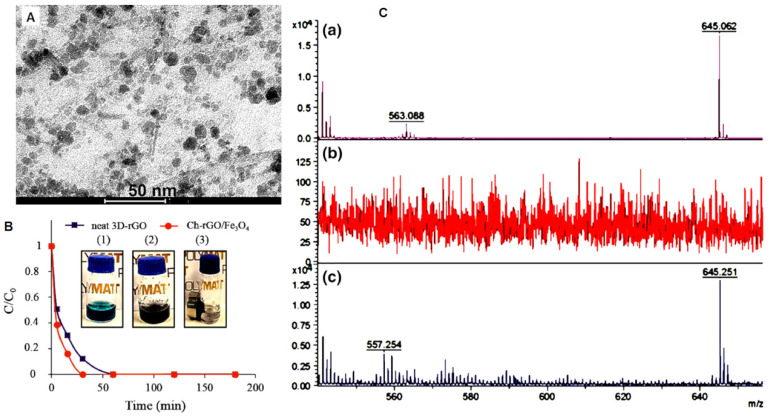
(**A**). TEM image of hybrid rGO/Fe_3_O_4_ aerogels. (**B**). Degradation kinetics of AG in aqueous solution by neat rGO and hybrid rGO/Fe_3_O_4_ aerogels. (**C**). MALDI-TOF-MS analysis results of AG aqueous solution (**a**) before treatment, (**b**) after treatment with hybrid rGO/Fe_3_O_4_ aerogels, and (**c**) after treatment with neat rGO aerogel. Adapted with permission from Ref. [54]. Copyright © 2021 Springer Nature.

The structure of the hybrid catalysts was investigated by TEM imaging [54]. Figure 4A shows that magnetic nanoparticles in irregular shape are distributed densely onto rGO platelets. It is worth mentioning that when AG degradation in aqueous solution was studied, the reaction was very fast and complete decolorization was achieved in less than 30 min (Figure 4B). Moreover, when the reaction pH was controlled to fall between the pKa values of the carboxylic acid groups on the rGO surface and the sulfonic acid functionalities of the AG dye, the electrostatic adsorption of the dye onto the 3D catalyst structure was significantly enhanced. This optimized pH range promoted strong ion–dipole interactions, facilitating the pre-concentration of dye molecules near the catalytic sites. Additionally, π–π stacking interactions between the graphenic domains of rGO and the extended aromatic rings of AG-25 further contributed to the adsorption mechanism. Under these optimized conditions, the Fenton degradation of AG-25 proceeded rapidly, achieving the full mineralization of the dye and its intermediate products in less than 30 min. This was confirmed by the MALDI–TOF–MS analysis of the reaction solution post-decolorization. To the best of the authors’ knowledge, this represents the most efficient catalytic performance reported to date for AG-25 degradation via a Fenton-like process [54].

## 6. Conclusions and Future Perspectives

The development of advanced catalytic systems for water treatment continues, driven by the urgent need for efficient, selective, and sustainable remediation technologies. Among the most promising approaches for water pollution remediation are advanced oxidation processes based on heterogeneous catalysts and processes such as photocatalysis, Fenton-like reactions, and enzyme-based biocatalysis, each offering distinct advantages and facing unique challenges. However, all of these processes offer the unique advantages of low-energy-cost processes and a lack of secondary pollution.

Graphene-based materials, particularly in their aerogel, hydrogel, and xerogel forms, have emerged as versatile platforms across all three domains. In Fenton and photo-Fenton systems, the incorporation of graphene aerogels significantly enhances pollutant degradation efficiency by improving electron transfer, radical generation, and catalyst stability. Tailoring material properties such as surface area, pore structure, and redox behavior through heteroatom doping and composite formation has allowed these systems to achieve complete dye degradation, often under mild conditions and with excellent reusability.

In the field of biocatalysis, graphene-derived hydrogels and xerogels have proven effective as inert, high-surface-area supports for enzyme or microorganism immobilization. Their tunable surface chemistry and hierarchical porosity not only facilitate strong enzyme–support interactions but also influence enzyme activity, stability, and long-term reusability. Composite strategies integrating polymers or natural reinforcements (e.g., lignin or PMMA-based latexes) further enhance the mechanical robustness and scalability of these platforms.

Collectively, these advances highlight the synergistic relationship between material design and catalytic performance. Whether used to accelerate oxidative degradation via Fenton chemistry or to support long-lived enzyme systems for pollutant breakdown, engineered graphene-based composites offer a powerful toolkit for next-generation water purification. Efforts should continue to address limitations in stability, handling, and cost, particularly through polymer integration and nanostructuring, which will be critical to transitioning these technologies from laboratory studies to scalable environmental applications. Future research will likely focus on optimizing the structural design and surface chemistry of rGO 3D porous monoliths, exploring synergistic effects with multifunctional nanocomposites, developing scalable fabrication techniques for real-world wastewater treatment applications, and incorporating life cycle assessment (LCA) studies and toxicity evaluations to ensure environmental safety and sustainability.

## Data Availability

No new data were created or analyzed in this study. Data sharing is not applicable to this article.

## References

[1] Lv D., Zhang Z., Zhang J., Zhang X., Liu L., Gong Y., Zhao J., Li Y. (2022). Modification of CI Pigment Red 146 with surfactants and graphene oxide. RSC Adv..

[2] Algarni T.S., Al-Mohaimeed A.M. (2022). Water purification by adsorption of pigments or pollutants via metaloxide. J. King Saud. Univ. Sci..

[3] Dadarwal B.K., Bahuguna A., Singh S.K., Bahuguna A., Sharma S. (2021). Physical Method of Wastewater Treatment—A Review. www.questjournals.org.

[4] Parvulescu V.I., Epron F., Garcia H., Granger P. (2022). Recent Progress and Prospects in Catalytic Water Treatment. Am. Chem. Soc..

[5] Wang L., Zhang Y., Qian J. (2021). Graphene aerogel-based catalysts in Fenton-like reactions for water decontamination: A short review. Chem. Eng. J. Adv..

[6] Guo J., Song G., Zhang X., Zhou M. (2023). Transition metal catalysts in the heterogeneous electro-Fenton process for organic wastewater treatment: A review. R. Soc. Chem..

[7] Orge C.A., Sousa J.P.S., Gonçalves F., Freire C., Órfão J.J.M., Pereira M.F.R. (2009). Development of novel mesoporous carbon materials for the catalytic ozonation of organic pollutants. Catal. Lett..

[8] Nawrocki J., Kasprzyk-Hordern B. (2010). The efficiency and mechanisms of catalytic ozonation. Appl. Catal. B Environ..

[9] Choi Y., Lee D., Hong S., Khan S., Darya B., Lee J.-Y., Chung J., Cho S.-H. (2020). Investigation of the synergistic effect of sonolysis and photocatalysis of titanium dioxide for organic dye degradation. Catalysts.

[10] Wang J., Lv Y., Zhang L., Liu B., Jiang R., Han G., Xu R., Zhang X. (2010). Sonocatalytic degradation of organic dyes and comparison of catalytic activities of CeO_2_/TiO_2_, SnO_2_/TiO_2_ and ZrO_2_/TiO_2_ composites under ultrasonic irradiation. Ultrason. Sonochem..

[11] Li W., Ye Q., Xia T., Zhao L., Yang M. (2022). Degradation of Organic Dyes Using the Ionizing Irradiation Process in the Presence of the CN/CD_3_/Fe_6_ Composite: Mechanistic Studies. ACS Omega.

[12] Alkhuraiji T.S., Alkhuraiji W.S. (2019). Detailed study of water radiolysis-based degradation of chloroorganic pollutants in aqueous solutions. J. Hazard. Mater..

[13] Santos A.S.G.G., Orge C.A., Pereira M.F.R., Soares O.S.G.P. (2024). Carbon Materials Application in Heterogeneous Catalysis for Water Treatment: A Pathway to Process Intensification. Catalysts.

[14] Long S., Wang H., He K., Zhou C., Zeng G., Lu Y., Cheng M., Song B., Yang Y., Wang Z. (2020). 3D graphene aerogel based photocatalysts: Synthesized, properties, and applications. Colloids Surf. A Physicochem. Eng. Asp..

[15] Gao B., Feng X., Zhang Y., Zhou Z., Wei J., Qiao R., Bi F., Liu N., Zhang X. (2024). Graphene-based aerogels in water and air treatment: A review. Chem. Eng. J..

[16] Politakos N., Barbarin I., Cantador L.S., Cecilia J.A., Mehravar E., Tomovska R. (2020). Graphene-Based Monolithic Nanostructures for CO_2_ Capture. Ind. Eng. Chem. Res..

[17] Rodriguez-Couto S., Arzac A., Leal G.P., Tomovska R. (2014). Reduced graphene oxide hydrogels and xerogels provide efficient platforms for immobilization and laccase production by Trametes pubescens. Biotechnol. J..

[18] Ormategui N., Veloso A., Leal G.P., Rodriguez-Couto S., Tomovska R. (2015). Design of Stable and Powerful Nanobiocatalysts, Based on Enzyme Laccase Immobilized on Self-Assembled 3D Graphene/Polymer Composite Hydrogels. ACS Appl. Mater. Interfaces.

[19] Garcia-Bordejé E., Benito A.M., Maser W.K. (2021). Graphene aerogels via hydrothermal gelation of graphene oxide colloids: Fine-tuning of its porous and chemical properties and catalytic applications. Adv. Colloid. Interface Sci..

[20] Wan W., Zhang F., Yu S., Zhang R., Zhou Y. (2016). Hydrothermal formation of graphene aerogel for oil sorption: The role of reducing agent, reaction time and temperature. New J. Chem..

[21] Nassar G., Daou E., Najjar R., Bassil M., Habchi R. (2021). A review on the current research on graphene-based aerogels and their applications. Carbon. Trends.

[22] Hutchison J.M., Mayer B.K., Vega M., Chacha W.E., Zilles J.L. (2021). Making Waves: Biocatalysis and Biosorption: Opportunities and Challenges Associated with a New Protein-Based Toolbox for Water and Wastewater Treatment. Water Res. X.

[23] Hutchison J.M., Poust S.K., Kumar M., Cropek D.M., MacAllister I.E., Arnett C.M., Zilles J.L. (2013). Perchlorate reduction using free and encapsulated azospira oryzae enzymes. Environ. Sci. Technol..

[24] Chen Y., Stemple B., Kumar M., Wei N. (2016). Cell Surface Display Fungal Laccase as a Renewable Biocatalyst for Degradation of Persistent Micropollutants Bisphenol A and Sulfamethoxazole. Environ. Sci. Technol..

[25] Efremenko E., Stepanov N., Senko O., Maslova O., Lyagin I., Aslanli A. (2023). Progressive Biocatalysts for the Treatment of Aqueous Systems Containing Pharmaceutical Pollutants. Life.

[26] Zdarta J., Jesionowski T., Pinelo M., Meyer A.S., Iqbal H.M., Bilal M., Nguyen L.N., Nghiem L.D. (2022). Free and immobilized biocatalysts for removing micropollutants from water and wastewater: Recent progress and challenges. Bioresour. Technol..

[27] Abdelhamid M.A.A., Khalifa H.O., Yoon H.J., Ki M.R., Pack S.P. (2024). Microbial Immobilized Enzyme Biocatalysts for Multipollutant Mitigation: Harnessing Nature’s Toolkit for Environmental Sustainability. Int. J. Mol. Sci..

[28] Wu D., Lv P., Feng Q., Jiang Y., Yang H., Alfred M., Wei Q. (2022). Biomass-derived nanocellulose aerogel enable highly efficient immobilization of laccase for the degradation of organic pollutants. Bioresour. Technol..

[29] Bilal M., Iqbal H.M.N. (2019). Naturally-derived biopolymers: Potential platforms for enzyme immobilization. Int. J. Biol. Macromol..

[30] Rodríguez-Couto S. (2011). Production of laccase and decolouration of the textile dye Remazol Brilliant Blue R in temporary immersion bioreactors. J. Hazard. Mater..

[31] Sebai W., Ahmad S., Marie-Pierre B., Boccheciampe A., Chaurand P., Levard C., Brun N., Galarneau A., Sanchez-Marcano J. (2022). Biocatalytic Elimination of Pharmaceutics Found in Water with Hierarchical Silica Monoliths in Continuous Flow. Front. Chem. Eng..

[32] Poznansky B., Thompson L.A., Warren S.A., Reeve H.A., Vincent K.A. (2020). Carbon as a Simple Support for Redox Biocatalysis in Continuous Flow. Org. Process Res. Dev..

[33] Obayomi K.S., Lau S.Y., Danquah M., Chiong T., Takeo M. (2022). Advances in graphene oxide based nanobiocatalytic technology for wastewater treatment. Environ. Nanotechnol. Monit. Manag..

[34] Zhou W., Zhang W., Cai Y. (2022). Enzyme-enhanced adsorption of laccase immobilized graphene oxide for micro-pollutant removal. Sep. Purif. Technol..

[35] Xie Y., Huang Q., Liu M., Wang K., Wan Q., Deng F., Lu L., Zhang X., Wei Y. (2015). Mussel inspired functionalization of carbon nanotubes for heavy metal ion removal. RSC Adv..

[36] Jiang S., Ren D., Wang Z., Zhang S., Zhang X., Chen W. (2022). Improved stability and promoted activity of laccase by One-Pot encapsulation with Cu (PABA) nanoarchitectonics and its application for removal of Azo dyes. Ecotoxicol. Environ. Saf..

[37] Bolibok P., Wiśniewski M., Roszek K., Terzyk A.P. (2017). Controlling enzymatic activity by immobilization on graphene oxide. Sci. Nat..

[38] Zhang H., Zhang X., Wang L., Wang B., Zeng X., Ren B., Yang X. (2023). Synthesis of a Lignin-Enhanced Graphene Aerogel for Lipase Immobilization. ACS Omega.

[39] Qiu B., Xing M., Zhang J. (2014). Mesoporous TiO_2_ nanocrystals grown in situ on graphene aerogels for high photocatalysis and lithium-ion batteries. J. Am. Chem. Soc..

[40] Trinh T.T.P.N.X., Giang N.T.H., Huong L.M., Thinh D.B., Dat N.M., Trinh D.N., Hai N.D., Oanh D.T.Y., Nam H.M., Phong M.T. (2022). Hydrothermal synthesis of titanium dioxide/graphene aerogel for photodegradation of methylene blue in aqueous solution. J. Sci. Adv. Mater. Devices.

[41] Li Y., Yang X., Yan S., Yang J., Jia X., Song H. (2024). Bioinspired Graphene Aerogels with Hybrid Wettability for Solar-Driven Purification of Complex Wastewater. ACS Appl. Mater. Interfaces.

[42] Zhang J.-J., Wu Y.-H., Mei J.-Y., Zheng G.-P., Yan T.-T., Zheng X.-C., Liu P., Guan X.-X. (2016). Synergetic adsorption and photocatalytic degradation of pollutants over 3D TiO_2_-graphene aerogel composites synthesized: Via a facile one-pot route. Photochem. Photobiol. Sci..

[43] Toshikj N., Politakos N., Veloso A., Román E.G.d.S., Cordero-Lanzac T., Qin Z., Leal G.P., Tomovska R. (2020). Visible Light Photocatalysts Based on Manganese Doped TiO_2_ Integrated Within Monolithic Reduced Graphene Oxide/Polymer Porous Monolith. ChemistrySelect.

[44] Buu T.T., Son V.H., Nam N.T.H., Hai N.D., Vuong H.-T., Quang L.T.K., Dat N.M., Lin T.H., Phong M.T., Hieu N.H. (2023). Three-dimensional ZnO–TiO_2_/graphene aerogel for water remediation: The screening studies of adsorption and photodegradation. Ceram. Int..

[45] Tomovska R., Politakos N., Barbarin I. (2022). Reduced Graphene Oxide with Semiconductor Containing Photocatalysts.

[46] Liu M., Ye Y., Ye J., Gao T., Wang D., Chen G., Song Z. (2023). Recent Advances of Magnetite (Fe_3_O_4_)-Based Magnetic Materials in Catalytic Applications. Magnetochemistry.

[47] Liu M., Ye Y., Xu L., Gao T., Zhong A., Song Z. (2023). Recent Advances in Nanoscale Zero-Valent Iron (nZVI)-Based Advanced Oxidation Processes (AOPs): Applications, Mechanisms, and Future Prospects. Nanomaterials.

[48] Liu M. (2024). Environmental remediation approaches by nanoscale zero valent iron (nZVI) based on its reductivity: A review. RSC Adv..

[49] Su S., Liu Y., He W., Tang X., Jin W., Zhao Y. (2019). A novel graphene oxide-carbon nanotubes anchored α-FeOOH hybrid activated persulfate system for enhanced degradation of Orange II. J. Environ. Sci..

[50] Yao T., Jia W., Feng Y., Zhang J., Lian Y., Wu J., Zhang X. (2019). Preparation of reduced graphene oxide nanosheet/FexOy/nitrogen-doped carbon layer aerogel as photo-Fenton catalyst with enhanced degradation activity and reusability. J. Hazard. Mater..

[51] Feng Y., Yao T., Yang Y., Zheng F., Chen P., Wu J., Xin B. (2018). One-step preparation of Fe_2_O_3_/reduced graphene oxide aerogel as heterogeneous Fenton-like catalyst for enhanced photo-degradation of organic dyes. ChemistrySelect.

[52] Qiu B., Deng Y., Du M., Xing M., Zhang J. (2016). Ultradispersed cobalt ferrite nanoparticles assembled in graphene aerogel for continuous photo-fenton reaction and enhanced lithium storage performance. Sci. Rep..

[53] Sadegh F., Politakos N., Román E.G.d.S., Sanz O., Perez-Miqueo I., Moya S.E., Tomovska R. (2020). A green synthesis of nanocatalysts based on reduced graphene oxide/magnetic nanoparticles for the degradation of Acid Red 1. RSC Adv..

[54] Sadegh F., Politakos N., de San Roman E.G., Sanz O., Modarresi-Alam A.R., Tomovska R. (2021). Toward enhanced catalytic activity of magnetic nanoparticles integrated into 3D reduced graphene oxide for heterogeneous Fenton organic dye degradation. Sci. Rep..

